# HOME-VISIT INVESTIGATION ON BODY SUPPORTING PROPERTIES OF DAILY PRODUCTS USING FORCE VECTOR FIELDS SENSOR

**DOI:** 10.1093/geroni/igae098.2716

**Published:** 2024-12-31

**Authors:** Ayano Nomura, Yoshifumi Nishida

**Affiliations:** Institute of Science Tokyo, Meguro, Tokyo, Japan; Tokyo Institute of Technology, Meguro City, Tokyo, Japan

## Abstract

Product accidents often occur due to poorly designed products that do not consider age-related functional changes. This study focuses on the inadequate ‘body-supporting properties’ of everyday products, which are recognized as a critical factor affecting independent living and fall prevention. To address this issue, we developed a portable system that measures and visualizes the vector fields of the hands’ body support, and we evaluated the body-supporting ability of everyday products in the home. A study was conducted to measure body-supporting movements using the developed system in 9 houses with 13 participants aged 69 to 86 years. The results of the study identified daily activities that involve body-supporting movements, such as putting on and taking off shoes and clothes, sitting and standing up, etc. The study also identified various everyday products, such as tables, footrests, fusuma (sliding doors or partitions used in traditional Japanese architecture), windows, TV racks, etc., that generate body-supporting forces. By using the proposed system to measure hand forces at multiple points, it is possible to investigate how the hands are used to hold everyday products and interact with the environment in daily life, as well as the direction in which force is applied to support the body. For example, research has shown that a body-supporting force of as little as 4 N in the horizontal direction is sufficient to maintain horizontal balance. This finding underscores the importance of considering body support in everyday environments for older people and provides valuable insights for future design improvements.

